# The causal role of gut microbiota in susceptibility of Long COVID: a Mendelian randomization study

**DOI:** 10.3389/fmicb.2024.1404673

**Published:** 2024-05-30

**Authors:** Zuming Li, Qinghua Xia, Jieni Feng, Xueru Chen, Yushi Wang, Xiaolei Ren, Siyi Wu, Rongyuan Yang, Jiqiang Li, Yuntao Liu, Yue Lu, Jiankun Chen

**Affiliations:** ^1^The Second Clinical Medical College, Guangzhou University of Chinese Medicine, Guangzhou, China; ^2^Qingyuan Hospital of Traditional Chinese Medicine, Qingyuan, China; ^3^The Second Affiliated Hospital (Guangdong Provincial Hospital of Chinese Medicine), Guangzhou University of Chinese Medicine, Guangzhou, Guangdong, China; ^4^Guangdong Provincial Key Laboratory of Research on Emergency in TCM, Guangzhou, China; ^5^State Key Laboratory of Traditional Chinese Medicine Syndrome, The Second Affiliated Hospital of Guangzhou University of Chinese Medicine, Guangzhou, Guangdong, China; ^6^State Key Laboratory of Dampness Syndrome of Chinese Medicine, The Second Affiliated Hospital of Guangzhou University of Chinese Medicine, Guangzhou, China; ^7^Guangdong Provincial Key Laboratory of Clinical Research on Traditional Chinese Medicine Syndrome, Guangzhou, China

**Keywords:** gut microbiota, Long COVID, Mendelian randomization, causality, genome-wide association study

## Abstract

**Background:**

Long COVID is a major challenge facing the public. Gut microbiota is closely related to Long COVID. However, the causal effects between gut microbiota and Long COVID remains unclear.

**Methods:**

Using summary statistics from Genome-Wide Association Studies (GWAS), Mendelian randomization (MR) analyses were performed to investigate the relationship between gut microbiota and Long COVID. The primary statistical method employed was Inverse Variance Weighted (IVW). Sensitivity analyses were then conducted to evaluate the reliability of the findings and account for potential confounding variables. Finally, a reverse MR analysis was conducted to examine potential associations between Long COVID and genetically predicted gut microbiota compositions.

**Results:**

There were 2 positive and 1 negative causal effect between gut microbiota and Long COVID. Meta-analysis results show that genus *Parasutterella* (OR = 1.145, 95%CI = 1.035 ∼ 1.266, *P* = 0.008) and genus *Oscillospira* (OR = 1.425, 95%CI = 1.235 ∼ 1.645, *P* < 0.001) significantly increased the risk of Long COVID. And genus *Eisenbergiella* (OR = 0.861, 95%CI = 0.785 ∼ 0.943, *P* = 0.001) significantly decreased the risk of Long COVID. Neither the pleiotropy nor the heterogeneity was observed. Reverse causal effect does not hold.

**Conclusion:**

Our research has provided genetic evidence that establishes multiple causal relationships between the gut microbiota and Long COVID, supporting the role of the gut microbiota in Long COVID. It is possible that different taxa play a role in the development of Long COVID. The causal relationships identified in this study require further investigation.

## Introduction

So far, 2019 coronavirus disease (COVID-19) has infected over 700 million people worldwide.^1^ Increasing evidence suggests that COVID-19 infection may lead to long-term complications in the pulmonary system as well as various extrapulmonary organ systems, such as the cardiovascular, neurological, gastrointestinal, and urogenital systems (Al-Aly et al., 2021; Bowe et al., 2023; Davis et al., 2023). Some patients suffer from persistent symptoms of Long COVID, unable to return to work within 1 year or even 2 years after infection, and repeatedly seek medical help, posing a certain burden on health and socio-economy (Huang et al., 2022; Parotto et al., 2023). In general, the symptoms of Long COVID significantly reduce the quality of life of patients and bring great trouble to their work and life. Therefore, it is imperative to study the potential risk factors of Long COVID and explore the prevention and treatment methods of Long COVID.

Gut microbiota is a collective term for various bacteria in the human gastrointestinal tract, which is a widely distributed and complex microbial community (Dickson et al., 2013). These gut microbiotas play important roles in physiological homeostasis and metabolism, including immune system development, vitamin production and nutrient absorption (O’Hara and Shanahan, 2006). More and more evidence suggest that COVID-19 infection can lead to sequelae in the lungs and extensive extrapulmonary organ systems (Bowe et al., 2023), which may be closely related to gut microbiota. Current research has shown that the gut microbiota of patients infected with COVID-19 is disrupted, and long-term disturbances occur after nucleic acid testing is negative. The composition of human gut microbiota may be associated with the risk of complications or persistent symptoms after several months of infection with COVID-19 (Liu Q. et al., 2022; Ren et al., 2023). By regulating intestinal microbiota, gastrointestinal symptoms of patients with “Long COVID” syndrome can be alleviated (Wang et al., 2022). In short, not only COVID-19 infection is related to gut microbiota disorder, but also after nucleic acid turns negative, Long COVID is closely related to gut microbiota disorder. Yet, these associations from traditional observational epidemiologic studies are susceptible to confounding factors and reverse causation bias, biasing experimental results.

Mendelian randomization (MR) is an epidemiological method that uses genetic variation as an instrumental variable for exposure to estimate the causal effect of exposure on outcomes and strengthens causal inference, overcoming the limitations of traditional observational study designs by using Mendelian laws, exploits the random assignment to genotypes at conception, making genotypes independent of potential confounders and also avoiding reverse causality (Smith et al., 2007). In recent years, two-sample MR studies have gradually been recognized by researchers, allowing data between genetic instrument variables and phenotypes, phenotypes and diseases to come from two different independent populations, improving the efficiency and statistical power of the study (Burgess et al., 2016; Gupta et al., 2017; Davies et al., 2018).

In conclusion, this study is the first systematic study of the relationship between Long COVID and gut microbial communities. We provide a new explanatory perspective on the complex causal relationship between Long COVID and gut microbiota.

## Materials and methods

### Study design

**FIGURE 1:**
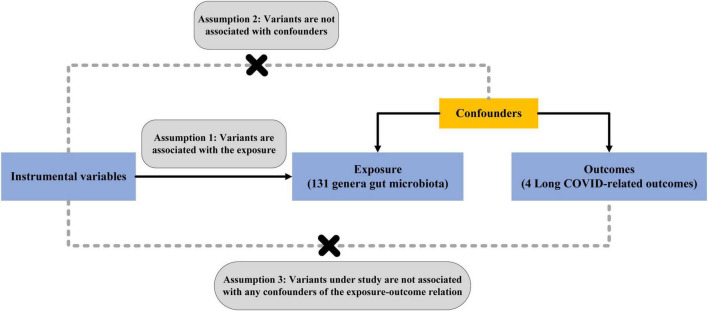
Schematic overview of Mendelian randomization design.

This study follows the STROBE-MR guidelines (Skrivankova et al., 2021) and follows the key principles of the Strengthening Epidemiological Observational Research Reporting (STROBE) guidelines (von Elm et al., 2007). In mendelian randomization, single-nucleotide poly-morphisms (SNPs) are defined as IVs. It is based on three core assumptions: (1) Genetic variation as instrumental variables is associated with gut microbiota; (2) Genetic variation is not associated with any unmeasured confounding factors related to Long COVID; (3) Genetic variation is only associated with Long COVID through the classification of gut microbiota and not through other pathways (Bowden and Holmes, 2019). The overview of the MR study design is shown in Figure 1. Finally, perform reverse MR analysis to mitigate the potential impact of Long COVID on the gut microbiota.

### Data source

The MiBioGen study represents the most extensive ethnically diverse genome-wide meta-analysis of the gut microbiome conducted thus far. The GWAS meta-analysis included a total of 18,340 individuals from 24 different cohorts. By focusing on the V4, V3-V4, and V1-V2 regions of the 16S rRNA gene, researchers were able to analyze the microbial composition of multiple cohorts, selecting 10,000 readings per cohort from all available microbiome datasets (Kurilshikov et al., 2021; Zhao et al., 2023). In this study, a comprehensive set of 131 microbial traits (131 genera) were incorporated as exposure.

We obtained a summary of four Long COVID related data from the latest published GWAS (Lammi et al., 2023). This study was published in the news section of the journal Nature (Ledford, 2023). This study used a total of four different case-control definitions to generate four types of GWAS, as shown below: (1) Long COVID cases after test-verified SARS-CoV-2 infection vs population controls (the strict case definition vs the broad control definition); (2) Long COVID within test-verified SARS-CoV-2 infection (the strict case definition vs the strict control definition); (3) Any Long COVID cases vs population controls (the broad case definition vs the broad control definition); (4) Long COVID within any SARS-CoV-2 infection (the broad case definition vs the strict control definition). Detailed information can be found in Supplementary Table 1.

The present study is a secondary analysis of publicly available GWAS summary statistics. Ethical approval was granted for each of the original GWAS studies. In addition, no individual-level data was used in this study. Therefore, no new ethical review board approval was required.

### Instrumental variables selection

First, we selected the SNPs with significant associations for gut microbiota (*P* < 1 × 10^–5^) (Cao et al., 2023; Dai et al., 2023). We excluded the SNPs with linkage disequilibrium (LD) in the analysis. This involved establishing a linkage disequilibrium (LD) threshold of r^2^ = 0.001 and implementing a clumping window of 10000kb (Myers et al., 2020). After matching the outcome, we removed palindromic SNPs.

F-statistic is utilized to quantify the association between genetic variants and exposure, with a higher F-statistic denoting a more robust instrument. A threshold of 10 is commonly acknowledged, as it signifies an instrument that accounts for a minimum of 10% of the variability in the exposure variable and exhibits a minimal likelihood of bias due to weak instrumentation. F-statistic for each instrumental variable (IV) is computed as F = (beta/se) ^2, and solely those IVs with an F-statistic exceeding 10 were included in the analysis (Burgess and Thompson, 2011a,b; Lv et al., 2023). Steiger Test is a new methodology that enhances the robustness of extracting instrumental variables (Hemani et al., 2017).

### MR statistical analysis

To assess the causal impact of gut microbiota on Long COVID, we conducted two-sample Mendelian randomization (MR) analysis for each outcome. The Inverse Variance Weighted (IVW) approach was employed as the primary analytical method. The IVW method initially computed ratio estimates for individual SNPs through the utilization of the Wald estimator and Delta method, subsequently amalgamating the calculated estimates from each SNP to derive the principal causal estimate (Burgess et al., 2013). If significant heterogeneity occurs, use a random effects model, and vice versa, use a fixed effects model (Chen et al., 2023). The results of MR analysis are represented by odds ratio (OR) and their respective 95% confidence intervals (CI). Weighted median (WM), MR-Egger and Weighted mode are auxiliary methods. The findings demonstrated statistical significance when the *P*-value of the IVW method was below 0.05, and IVW, Weighted median, MR-Egger, Weighted mode and conditional MR estimates exhibited consistent directional effects (Ji et al., 2024). To evaluate bi-directional causation effects between gut microbiota and Long COVID outcomes, we used Long COVID as “exposure” and gut microbiota as “outcome”. We selected the SNPs significantly associated with Long COVID outcomes (*P* < 5 × 10^–8^) as IVs. Since the result of IVW method is susceptible to the influences of valid instruments and potential pleiotropic effects, we performed sensitivity analyses to assess the robustness of the association. We performed Cochran’s Q test to evaluate the heterogeneity of each SNP (Cohen et al., 2015). Leave-one-out analysis was performed to evaluate if each SNP could affect the results (Burgess and Thompson, 2017). In addition, we used MR-PRESSO and MR-Egger regression to test the potential horizontal pleiotropy effect. MR-PRESSO was used to detect significant outliers and to correct the horizontal plural effect by removing outliers (Verbanck et al., 2018). All analyses were carried out using R (v4.3.0) statistical software. MR analysis was performed using the “TwoSampleMR” package. The “MR_PRESSO” package was used for multiplicity tests (Ong and MacGregor, 2019).

## Results

**FIGURE 2:**
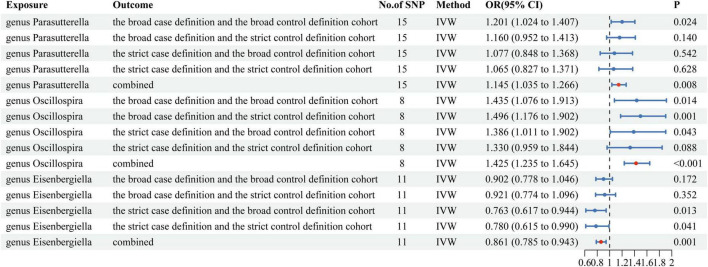
Mendelian randomization results of causal effects between gut microbiotas and Long COVID.

Our research findings suggest that there is a causal effect between three gut microbiotas at the level of genera and one or more long-term COVID-19 outcome data (Supplementary Table 2). Then, we conducted a multi-outcome meta-analysis of the three gut microbiotas. As shown in Figure 2, Genetic prediction of two gut microbiotas (genus *Parasutterella* and genus *Oscillospira*) was associated with an increased risk of Long COVID. Genetic prediction of one gut microbiota (genus *Eisenbergiella*) was associated with a decreased risk of Long COVID. The genus *Parasutterella* (OR = 1.145, 95%CI = 1.035 ∼ 1.266, *P* = 0.008) and genus *Oscillospira* (OR = 1.425, 95%CI = 1.235 ∼ 1.645, *P* < 0.001) significantly increased the risk of Long COVID. And genus *Eisenbergiella* (OR = 0.861, 95%CI = 0.785 ∼ 0.943, *P* = 0.001) significantly decreased the risk of Long COVID. These observed associations were directionally consistent with MR sensitivity analyses, including weighted median, weighted mode and MR-Egger methods (Supplementary Table 3). However, MR-Egger causal estimates between genus *Parasutterella* and Long COVID (the strict case definition vs the broad control definition cohort as outcome) diverged from the IVW method, and Weighted mode causal estimates between genus *Parasutterella* and Long COVID (the broad case definition vs the strict control definition cohort as outcome) diverged from the IVW method, it may had been due to poor precision. Neither the pleiotropy nor the heterogeneity was observed (Supplementary Tables 4–6). The findings from the “leave-one-out” analysis provide evidence supporting the reliability of MR analysis (Supplementary Figure 1 and Supplementary Table 7). Funnel plots and scatter plots depicted the collective impact of gut microbiota on Long COVID-related outcomes (Supplementary Figures 2, 3). A *P*-value threshold of less than 5 × 10^–8^ is used as a criterion for identifying SNPs that exhibit significant correlation with Long COVID. However, we did not obtain enough statistically significant SNPs, which led us to conclude that the existence of reverse causal effects has not been confirmed.

## Discussion

Given the existing body of knowledge, this study serves as a groundbreaking endeavor in investigating the causal relationships between gut microbiota and Long COVID. In the present investigation, a Mendelian randomization analysis was conducted to evaluate the potential causal relationship between gut microbiota and the susceptibility to Long COVID. The results of this study suggest that variations in the abundance of host-genetic-driven genera *Parasutterella*, *Oscillospira* and *Eisenbergiella* may contribute to Long COVID, which advances our understanding of the role of gut microbiota in Long COVID.

A prior cohort study conducted in two hospitals revealed a potential correlation between the gut microbiome and the severity of COVID-19 (Yeoh et al., 2021). Substantial and enduring alterations in the fecal microbiome were observed in COVID-19 patients in comparison to those who were healthy (Zuo et al., 2020). Further prospective investigations have indicated that the diversity of gut microbiota failed to revert to pre-infection levels following SARS-CoV-2 infection, with alterations in gut microbiota being closely associated with long-term complications post COVID-19 (Chen et al., 2022; Liu Q. et al., 2022). All indications indicate a close correlation between gut microbiota and COVID-19 and Long COVID. Based on previous research, our findings suggest that changes in the abundance of gut microbiota composition may have a causal impact on the susceptibility of Post COVID-19 Syndromes. The changes in gut microbiota abundance may help explain various Post COVID-19 Syndromes. Therefore, for Long COVID patients, maintaining a balance of gut microbiota is crucial.

Our main finding is a causal relationship between gut microbiota and Long COVID. This has a significant impact on the population at large, and the long-term impact of Long COVID exceeds initial estimates. While the exact cause of Long COVID remains unclear, existing studies indicate that COVID-19 infection may lead to its onset and progression through immune system influence, persistence of the virus and inflammation (Song et al., 2021). Gut microbiota may play an important role in this. Gut microbiota regulates body’s immune response. In the case of gut microbiota disorder, the regulation of nature is lost. This paves the way for overreactions, which can persist long after an acute infection ends and affect many different parts of the body (Zhang et al., 2023). Disturbance of the gut microbiota is both a risk factor for infection with COVID-19 and is often the result of infection (Song et al., 2023). ACE-2 receptors that bind to the COVID-19 can also disrupt the composition of the gut microbiota by reducing gut microbiotal diversity (Zhang et al., 2023). This destruction may reduce the levels of beneficial gut microbiota and their important metabolites, short-chain fatty acids, which may lead to immune system disorders and overreactions, leading to symptoms of Long COVID (de Oliveira et al., 2021). Because gut microbiota play an important role not only in the immune response, but also in many aspects of physiology, such as hormone and vitamin synthesis, detoxification of harmful compounds, digestion and absorption of nutrients, etc., coupled with the recognized role of the gut-brain axis in cognition, emotion, memory and neurological function, changes in the health and balance of the gut microbiota caused by viral infection can translate into systemic symptoms (Liu Q. et al., 2022; Wang et al., 2023). For the rehabilitation management and treatment of patients with chronic COVID-19, measures should be taken to maintain the stability of gut microbiota to promote patient recovery. However, further research is still needed to investigate the interaction between gut microbiota and Post-COVID-19 Syndrome, as well as potential effective interventions targeting microbiota for Post-COVID-19 Syndrome.

This study found that genetic prediction of two gut microbiotas (genus *Parasutterella* and genus *Oscillospira*) was associated with an increased risk of Long COVID. *Parasutterella* is a genus of Gram-negative, coccobacilli, strictly anaerobic, non-s pore forming bacteria from the *Proteobacteria* phylum, *Betaproteobacteria* class and the *Sutterellaceae* family (Nagai et al., 2009; Morotomi et al., 2011). Research has shown that *Parasutterella* is associated with intestinal mucosal inflammatory response (Chen et al., 2018) and systemic metabolic abnormalities (Xiao et al., 2020), which may indicate its role in the development of systemic low-grade metabolic inflammation caused by ecological imbalance. Current research suggests that long-term inflammation and metabolic disorder may be important factors for Long COVID. Moreover, a recent study found that the abundance of *Parasutterella* is positively correlated with BMI and type 2 diabetes, and positively correlated with carbohydrate intake in diet (Henneke et al., 2022), which indicates the role of *Parasutterella* in human type 2 diabetes and obesity, and the relationship with L-cysteine may be related to the development of type 2 diabetes, and it is related to the fatty acid biosynthesis pathway that leads to weight gain during the development of obesity due to a carbohydrate rich diet. A meta-analysis showed that compared with non COVID-19, COVID-19 patients had an overall risk of developing diabetes (type 1 or type 2) of 62% (Zhang T. et al., 2022). Canadian scholars based on the study of more than 600000 people further confirmed that compared with non COVID-19, COVID-19 infected people have an increased risk of diabetes of about 50%, which is equivalent to at least 3–5% of diabetes overload at the population level (Naveed et al., 2023). Therefore, the increased abundance of *Parasutterella* may mediate the risk of obesity and diabetes after COVID-19. *Oscillospira* is an understudied genus of anaerobic bacteria in the *Clostridium* community, belonging to the phylum *Firmicutes* and the family *Ruminococcaceae* (Konikoff and Gophna, 2016). Research has shown that *Oscillospira* is linked to adiposity, metabolic dysfunction, gut inflammation, and serum triglycerides in both mice and humans (Guadagnini et al., 2019). Chen et al. (2020) further demonstrated that *Oscillospira* plays a significant role in human health, as its abundance is positively associated with high-density lipoprotein levels, microbial diversity, and sleep duration, while inversely correlated with blood pressure, fasting blood glucose levels, uric acid levels, triglyceride levels, and Bristol stool type. In addition, *Oscillospira* levels were higher in HFD mice compared to control mice, leading to increased production of short-chain fatty acid (SCFA) like acetate, propionate, and butyrate (Liu et al., 2021). The SCFAs butyrate supplement demonstrated efficacy in mitigating symptoms associated with low estrogen levels, including increased osteoclastogenesis and bone loss in estrogen-deficient mice following ovariectomy (Liu L. et al., 2022; Zhang Y. W. et al., 2022). Further research is needed to fully understand the relationship between *Oscillospira*, host health, SCFAs production, and estrogen levels. Our hypothesis suggests that *Oscillospira* may play a role in the development of Long COVID in cases where there is gut dysbiosis and estrogen imbalance. We also found that genetic prediction of genus *Eisenbergiella* was associated with a decreased risk of Long COVID. *Eisenbergiella* has been identified as harboring species capable of metabolizing the SCFA butyrate derived from dietary carbohydrates (Amir et al., 2014; Togo et al., 2016; Cammann et al., 2023). Butyrate, a prominent SCFA metabolite in the colon, has been suggested as a key mediator in the inflammatory response within the colon. In addition to its anti-inflammatory characteristics, butyrate plays a crucial role in the maintenance of tight junctions, which serve to prevent dysbiotic gut permeability(Kelly et al., 2015; Silva et al., 2020). More research is needed to fully understand the connection between gut microbiota and disease, especially in infection-related diseases.

The development and management of Long COVID are multifactorial, with this study specifically examining microecological differences as a component of understanding the condition. It is essential to acknowledge the complexity of Long COVID and the need for a comprehensive evaluation of various factors to gain a thorough understanding. This study is the first to reveal a potential causal effect between three gut microbiotas (genera *Parasutterella*, *Oscillospira* and *Eisenbergiella*) and Long COVID, which has profound implications for public health and the management of individual patients with Long COVID. This finding may help diagnose Long COVID more accurately. Based on the presence of three gut microbiotas, COVID-19 patients can be screened and assessed for risk, so that preventive measures can be taken in advance to reduce the incidence of Long COVID (Peng et al., 2024). In addition, this research also contributes to a deep understanding of the pathogenesis and pathological process of Long COVID, providing scientific basis for formulating targeted public health policies and prevention and control measures. Alleviate symptoms of Long COVID or facilitate recovery by adjusting gut microbiota balance. Interventions targeting targeted gut microbiota for more effective treatment of Long COVID (Cai et al., 2022).

Using a MR design with genetic variants as IVs allows for exploring causal relationships between exposures and outcomes, while minimizing potential confounding factors present in observational studies. Our study benefits from using the largest publicly available GWAS datasets, increasing statistical power and accuracy in detecting causal effects. Microecological studies often have conflicting results due to the lack of standardized sampling methods and longitudinal studies. Confounding factors and reverse causality can complicate observational studies. We aim to improve understanding of how gut microbiota and Long COVID are connected using a strong genetic approach. This study still has exploratory significance.

There are some limitations to our study. First, the gut microbiome is influenced by many small-effect variants, so we used a less strict *p*-value threshold to account for this complexity and increase statistical power. Second, while the majority of participants in the GWAS data were of European descent, a small non-European population was also included, potentially impacting the accuracy of our results. In addition, it remains to be verified whether there are genetic differences between other races, countries and regions. It is hoped that future studies will include more diverse populations to obtain broader and more convincing results. Third, even though pleiotropy and heterogeneity were not observed in this study, further studies are needed for rigor and completeness of the results. Pleiotropy is usually due to genetic variation having multiple biological functions, which may lead to genetic variation affecting outcomes through non-attention pathways, thus violating Mendelian randomization assumptions. It is necessary to understand how the marker of the organism studied behaves in other studies and to assess whether it has the potential for pleiotropy. Heterogeneity may result from factors such as differences between individuals in the sample, differences in study design or data analysis methods. To explore heterogeneity more fully, subsequent investigators can perform more detailed stratified analyses of samples to explore whether heterogeneity exists between subgroups. In addition, consider using more sophisticated statistical models or methods, such as mixed effects models or sensitivity analyses, to assess the impact of heterogeneity on study results. Our Mendelian randomization study was unable to obtain individual-level data, such as sex, age and disease severity, which limited the depth of our analysis. Finally, although we have demonstrated a bidirectional causal relationship between gut microbiota and Long COVID, the underlying mechanisms are not known. The mechanism of interaction between gut microbiota and Long COVID needs to be further investigated in depth.

## Conclusion

In summary, our bidirectional Mendelian randomization study clearly demonstrates a causal relationship between gut microbiota and Long COVID, while the reverse causality hypothesis did not hold. To gain a more nuanced understanding of the observed association between gut microbiota and Long COVID, future studies should focus on potential mechanistic pathways, while also attempting to adjust for potential confounders such as diet, lifestyle, and medications, provided that these factors may have a greater impact on Long COVID. Our work is an important step forward in explaining the relationship between gut microbiota and Long COVID, but more microbiological and clinical studies are still needed to validate and expand our findings. We hope that our research can contribute to the diagnosis, treatment, and drug development of Long COVID.

## Data availability statement

All data used in the present study were obtained from genome wide association study summary statistics which were publicly released by genetic consortia.

The summary data of genome-wide association studies (GWAS) related to gut microbiota can be obtained from the MiBioGen consortium at https://mibiogen.gcc.rug.nl/.

The summary data of genome-wide association studies (GWAS) related to Long COVID can be obtained from the Long COVID Host Genetics Initiative. A total of four different case-control definitions to generate four types of GWAS, as shown below.

(1) Strict case definition (Long COVID after test-verified SARS-CoV-2 infection) vs broad control definition (population control): https://my.locuszoom.org/gwas/192226/?token=09a18cf9138243db9cdf79ff6930fdf8
(2) Broad case definition (Long COVID after any SARS-CoV-2 infection) vs broad control definition: https://my.locus zoom.org/gwas/826733/?token=c7274597af504bf3811de6d74 2921bc8
(3) Strict case definition vs strict control definition (individuals that had SARS-CoV-2 but did not develop Long COVID): https://my.locuszoom.org/gwas/793752/?token=0dc986619af14b6e8a564c580d3220b4
(4) Broad case definition vs strict control definition: https://my.locuszoom.org/gwas/91854/?token=723e672edf13478e817ca44b56c0c068.

## Ethics statement

The present study is a secondary analysis of publicly available data. Ethical approval was granted for each of the original GWAS studies. In addition, no individual-level data were used in this study. Therefore, no new ethical review board approval was required.

## Author contributions

ZL: Writing – review and editing, Writing – original draft, Software, Methodology, Investigation, Formal analysis, Data curation. QX: Writing – review and editing, Visualization, Validation, Methodology, Formal analysis, Writing – original draft. JF: Data curation, Writing – original draft, Investigation. XC: Writing – original draft, Methodology, Investigation. YW: Investigation, Writing – review and editing, Methodology, Data curation. XR: Formal analysis, Data curation, Writing – review and editing, Methodology. SW: Writing – review and editing, Methodology. RY: Writing – review and editing, Visualization, Validation, Supervision, Resources, Funding acquisition. JL: Writing – review and editing, Visualization, Validation, Supervision, Resources, Project administration, Funding acquisition. YTL: Project administration, Writing – review and editing, Visualization, Validation, Supervision, Resources, Funding acquisition. YL: Writing – review and editing, Writing – original draft, Visualization, Validation, Supervision, Resources, Project administration, Funding acquisition. JC: Writing – review and editing, Visualization, Validation, Supervision, Resources, Funding acquisition.

## Footnotes

1
https://covid19.who.int/
