# Supplementary material for: The causal role of gut microbiota in susceptibility of Long COVID: a Mendelian randomization study
Source: Front Microbiol. 2024 May 30;15:1404673. doi: 10.3389/fmicb.2024.1404673 (PMC11169722; doi:10.3389/fmicb.2024.1404673)
Supplement: Supplementary file 1 [file Data_Sheet_1.DOCX]

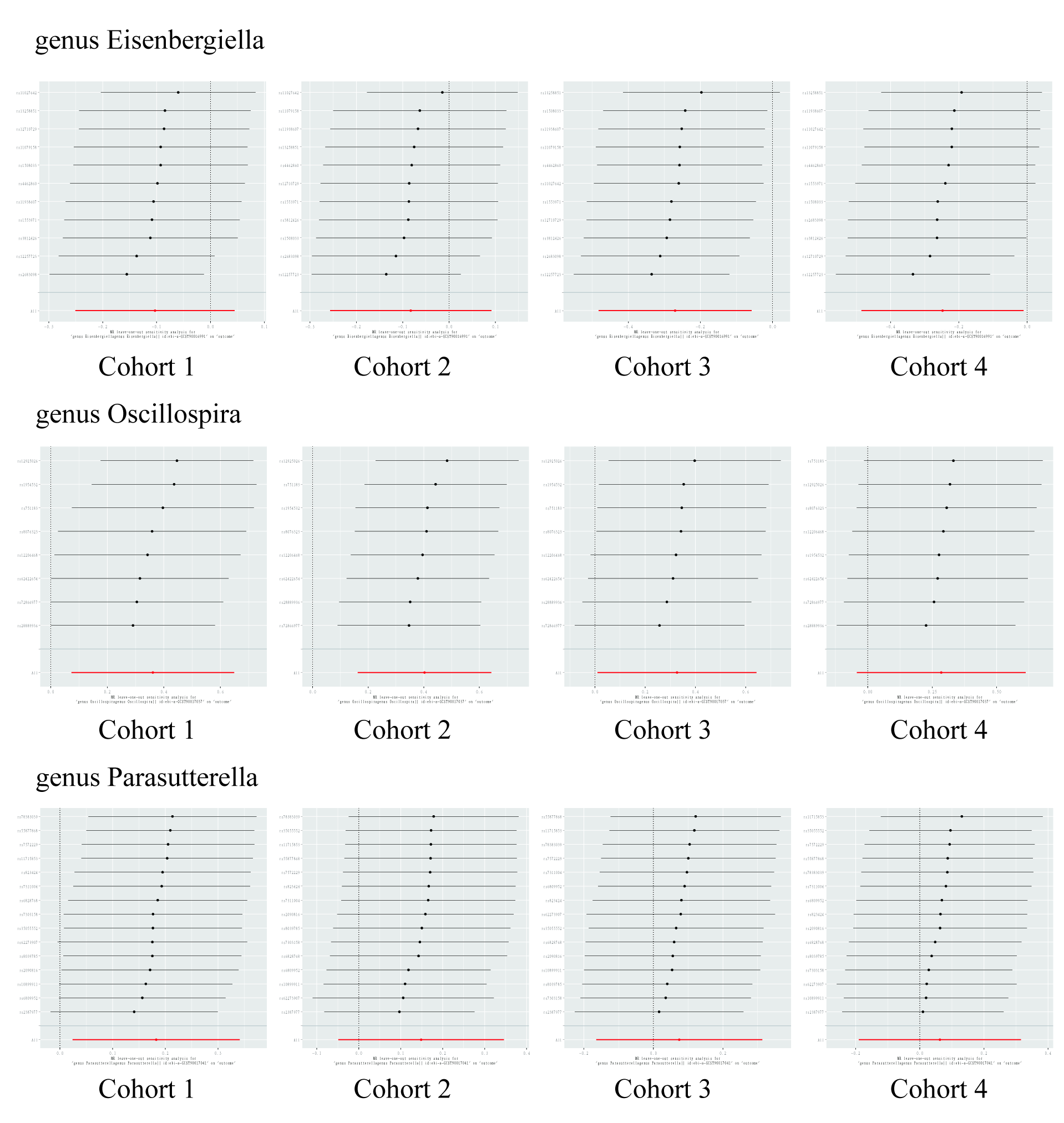


**Supplementary Figure 1** Leave-one-out analysis graphically depicts the causal associations between the gut microbiota and Long COVID (Cohort 1: the broad case definition and the broad control definition cohort; Cohort 2: the broad case definition and the strict control definition cohort; Cohort 3: the strict case definition and the broad control definition cohort; Cohort 4: the strict case definition and the strict control definition cohort).


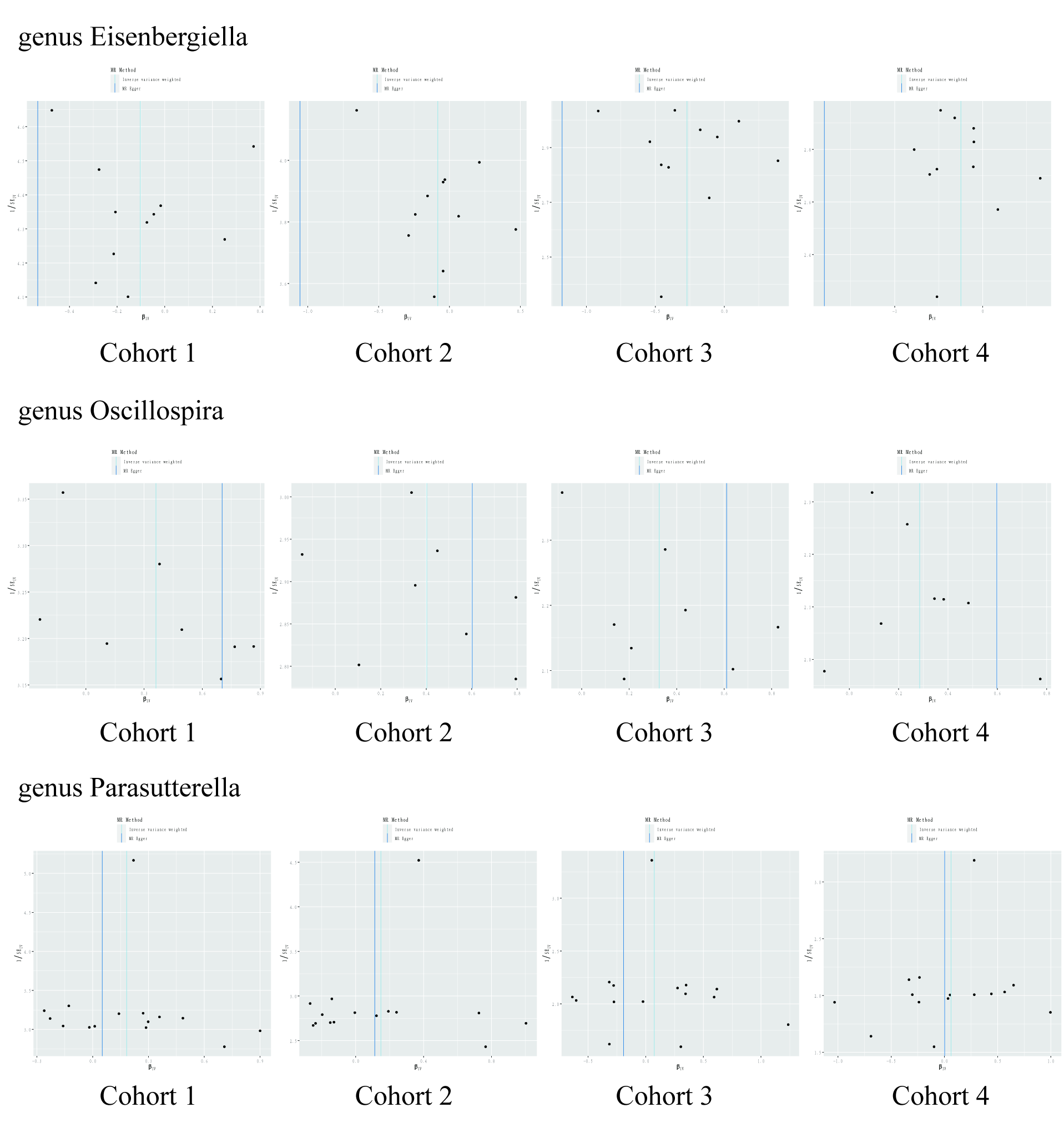


**Supplementary Figure 2** Funnel plot delineates the causal associations between the gut microbiota and Long COVID.


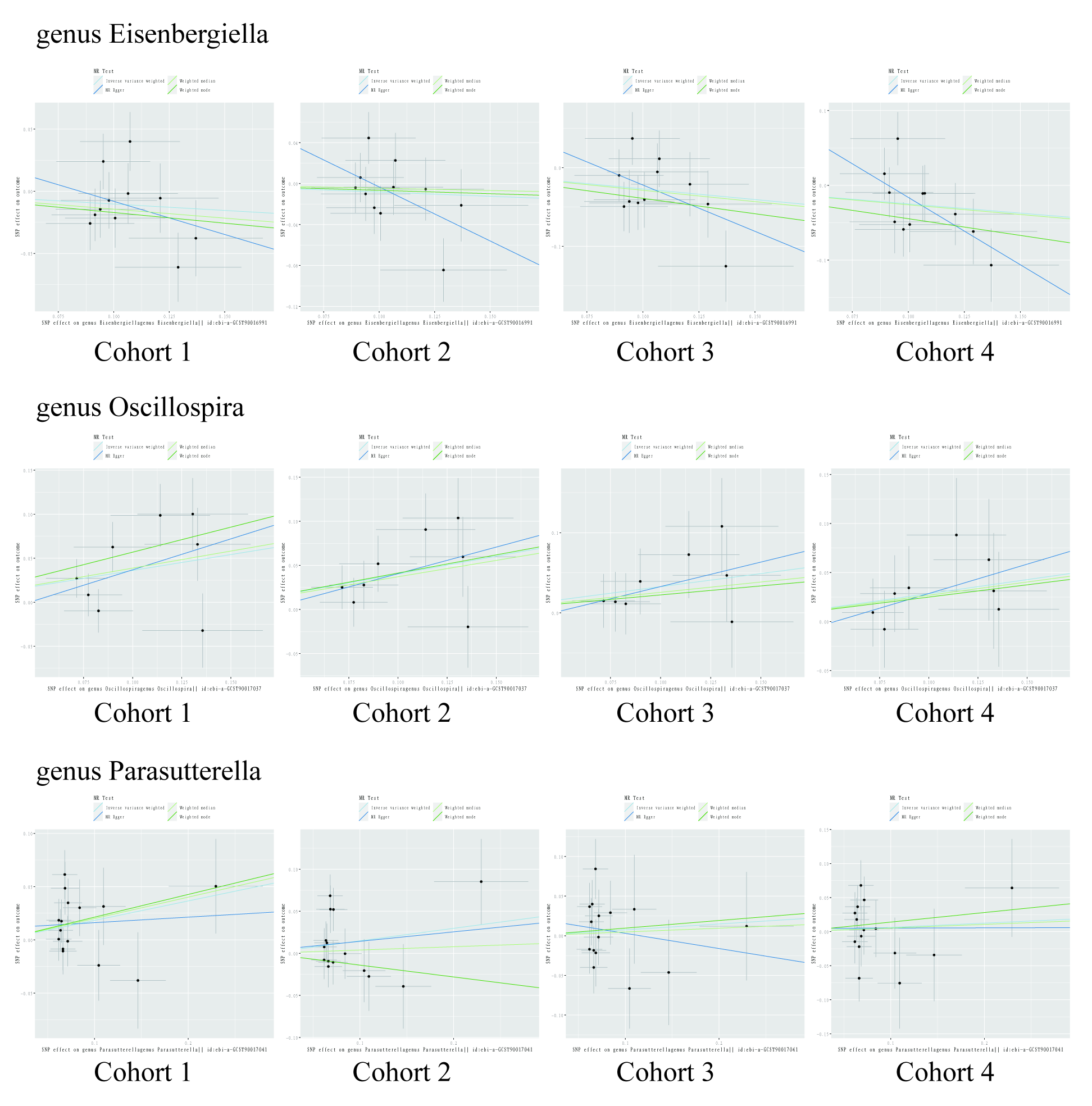


**Supplementary Figure 3** Scatter plot illustrates the causal associations between the gut microbiota and Long COVID.
